# Bio-Obturation for Internal Root Resorption in Contralateral Mandibular Molars: A Five-Year Case Study

**DOI:** 10.7759/cureus.76056

**Published:** 2024-12-20

**Authors:** Saeed Asgary

**Affiliations:** 1 Endodontics, Iranian Center for Endodontic Research, Research Institute of Dental Sciences, Shahid Beheshti University of Medical Sciences, Tehran, IRN

**Keywords:** apical periodontitis, calcium-enriched mixture, calcium silicate cement, cem cement, internal root resorption, minimally invasive dentistry

## Abstract

Internal root resorption (IRR) is a rare but complex condition characterized by progressive destruction of the internal dentin walls, typically resulting from chronic pulp inflammation, trauma, or infection. Managing apical IRR, particularly in teeth with extensive apical lesions, presents significant challenges due to the limitations of traditional root canal treatment (RCT) and obturation techniques. This report discusses the nonsurgical management of two contralateral mandibular first molars in a 49-year-old male patient, both exhibiting apical IRR and large endodontic lesions. Despite the previous RCT, the teeth continued to show clinical symptoms and radiographic evidence of persistent apical lesions and resorptive defects. The patient elected nonsurgical retreatment with bio-obturation using endodontic biomaterials. After cleaning, disinfecting, and preparing the root canals, the teeth were obturated with a calcium-enriched mixture cement. Within one week, the patient’s symptoms resolved, and a radiographic follow-up at five years revealed complete healing of the apical lesions. This case underscores the effectiveness of bio-obturation using endodontic biomaterials in treating apical IRR with large lesions, providing a nonsurgical solution that preserves natural tooth structure, promotes healing, and minimizes the risk of reinfection. Bio-obturation with endodontic biomaterials should be considered a viable treatment option for IRR, especially in cases with extensive apical lesions, and further studies are warranted to evaluate its long-term efficacy in more complex cases.

## Introduction

Among the 11 different types of tooth resorption, internal root resorption (IRR) is a pathological condition where the internal dentin walls of the root are progressively destroyed, creating a radiolucent area within the root canal [1]. This condition often creates irregular cavities within the root canal, presenting a significant challenge in achieving adequate debridement, filling, and sealing during endodontic treatment. Various factors, including traumatic injury, inflammation/infection, and orthodontic treatment, can trigger this process [2]. Clinically, IRR is associated with chronic pulp inflammation and can compromise the structural integrity of the tooth. Although the condition is recognized by most dentists, its etiology and pathogenesis are only partially understood, and it remains rare, particularly in the apical regions. IRR can be challenging to distinguish from cervical invasive or external inflammatory root resorption, often leading to misdiagnosis. Unlike external resorption, IRR typically stops when the entire pulp tissue becomes necrotic due to infection or extirpated during endodontic treatment. When diagnosed early and managed properly, the prognosis for teeth with IRR is generally favorable [3]. However, if the resorption leads to perforation or extensive damage, the tooth’s structural integrity may be compromised, complicating both infection control and restoration.

The management of apical internal root resorption can be complex, particularly in teeth that have previously undergone root canal treatment (RCT) and developed large apical lesions. Various treatment strategies have been proposed, including surgical intervention, nonsurgical orthograde endodontic retreatment, and regenerative endodontic procedures [4]. Traditional root canal obturation methods, such as gutta-percha with sealers, may not always provide a reliable seal in the presence of resorptive defects, especially in cases with large apical lesions or resorption cavities. To address this challenge, techniques such as warm vertical condensation, continuous wave of condensation, thermo-injectable gutta-percha, carrier-based obturation, and hybrid methods are commonly employed to improve the adaptation to complex canal anatomies, including resorptive defects [5]. However, in recent years, there has been a growing trend toward using biomaterials, which are increasingly recommended for their superior sealing ability and biocompatibility, offering more promising long-term outcomes [2,5].

One such innovative approach is bio-obturation, which utilizes biocompatible endodontic materials to optimize root canal filling, enhance sealing properties, and promote the healing/regeneration of surrounding tissues. Bio-obturation materials, such as mineral trioxide aggregate (MTA) and calcium-enriched mixture (CEM) cement, have demonstrated superior sealing abilities and exceptional biocompatibility, making them particularly effective for managing resorptive lesions [6,7]. MTA and CEM cement are bioceramic materials known for their excellent biocompatibility and sealing properties. They are highly effective in promoting tissue healing/regeneration, making them particularly useful in endodontic procedures such as bio-obturation. Bio-obturation refers to using biomaterials to fill the root canal system, enhance sealing, and promote surrounding tissue regeneration. The release of bioactive ions from biomaterials stimulates bioseal and tissue healing and supports the regeneration of periapical tissues, offering a nonsurgical approach to managing resorptive lesions in complex cases of root resorption. These biomaterials not only provide a tight seal within the root canal system but also promote the healing of periapical tissues through the release of bioactive ions, which stimulate tissue regeneration and enhance the body’s natural healing response. This approach offers a nonsurgical, internal treatment option, which is especially advantageous in complex cases of root resorption, where maintaining the tooth structure and promoting tissue regeneration are essential for long-term success.

The present study aimed to present the successful nonsurgical management of two contralateral mandibular first molars with apical internal root resorption and large apical lesions using the bio-obturation technique. This report also provides a detailed description of the clinical procedures, materials used, and long-term (five-year) follow-up results, demonstrating the effectiveness of bio-obturation in treating rare and challenging endodontic cases.

## Case presentation

A 49-year-old male patient presented with persistent discomfort in the lower left posterior region, specifically around the first molars (teeth 36, 46). The patient reported discomfort primarily during chewing. He had a history of RCT in both lower first molars approximately five years prior, which had been performed at another dental facility. Both teeth were restored with porcelain-fused-to-metal (PFM) crowns. Clinical examination revealed no significant mobility or deep probing, but there was mild tenderness to percussion and palpation in the apical regions of both lower first molars. The patient had a history of multiple dental caries, which were treated with restorations and crowns over the years. He has no significant medical history of note. No laboratory examinations were needed/conducted.

**Figure 1:**
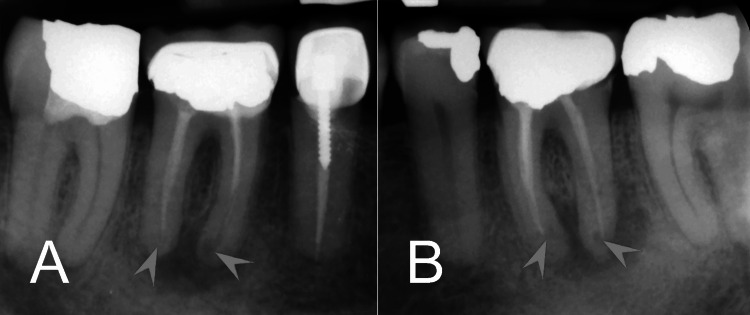
Preoperative periapical radiographs. (A) Tooth #46 showing underfilled root canals with short fillings, a large apical radiolucent lesion, and signs of IRR in the apical portions of both mesial and distal roots (arrowheads). (B) Tooth #36 displaying similar findings. IRR: internal root resorption

Periapical radiographs were obtained for both affected teeth. Radiographic images revealed poor root canal treatment with short filling lengths, leaving the canals underfilled and short of the apex (Figures 1A, 1B). Large apical radiolucent lesions were noted, extending beyond the root apices of both teeth. Additionally, both molars exhibited signs of IRR localized to the apical portions of all roots. The resorptive defects were relatively small, involving nonsignificant destruction of the internal dentin walls along with associated chronic inflammatory changes of the roots. The endodontic diagnosis was of endodontically treated teeth with IRR and symptomatic apical periodontitis, characterized by periapical lesions and resorptive defects.

The patient was presented with several treatment options, including extraction and complex implant replacement, surgical endodontic retreatment, or nonsurgical retreatment using biomaterials. After considering the available options, the patient expressed a preference for attempting to save the teeth, if possible, and opted for nonsurgical retreatment utilizing biocompatible biomaterials. Written informed consent was obtained from the patient.

For the first visit, the patient opted to retain the existing PFM crown, which was considered in good condition. The access cavity was carefully prepared through the PFM crown of tooth #36 using sharp tungsten carbide burs, preserving the integrity of the restoration. The working length was determined to be 21 mm, using an electronic apex locator for accuracy. Upon retreatment, the previously placed obturating material (gutta-percha and sealer) was meticulously removed using a heated instrument, Gates-Glidden burs (#2-5), and hand files (K-file; Tochigi-Ken, Japan: Mani Inc.), ensuring complete removal from the canal walls and resorptive defect areas while simultaneously preparing and enlarging the root canal system. The canals were subsequently re-evaluated to assess the extent of the resorptive defect. During this evaluation, mild bleeding was observed from the canals, which was effectively managed through additional chemomechanical instrumentation with 5.25% sodium hypochlorite. Furthermore, residual debris within the resorptive defect was identified and thoroughly flushed out with 5.25% sodium hypochlorite irrigation, ensuring effective disinfection of the canals. The resorptive defect was accessible without significant difficulty, and the decision was made to proceed with a single-visit retreatment based on the patient's preference and the absence of any complications that would necessitate additional visits. Saline rinsing was then performed to neutralize the irrigant, followed by a thorough drying of the root canals using paper points to facilitate optimal access to the resorptive defect.

**Figure 2:**
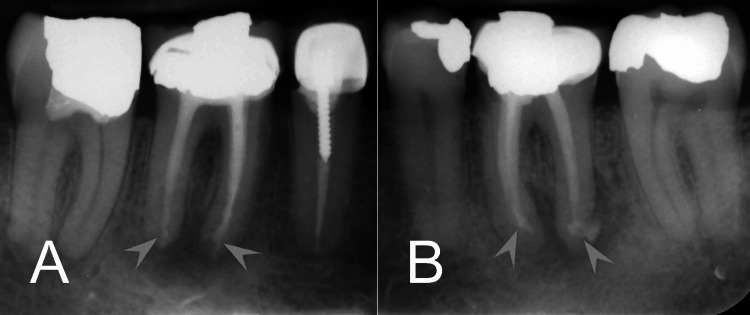
Immediate postoperative radiographs after bio-obturation with CEM cement. (A) Tooth #46 demonstrating well-adapted root canal filling with no visible gaps or complications. The IRRs were completely filled/sealed with bio-obturation (arrowheads) (B) Tooth #36 showing similarly successful bio-obturation with proper sealing of the root canal system and resorptive defects (arrowheads). CEM: calcium-enriched mixture; IRR: internal root resorption

Given the presence of IRR, both the resorptive defect and the root canal system were obturated with CEM cement (Tehran, Iran: Bioniquedent). The CEM cement was introduced using K-files and hand pluggers of various sizes to ensure adequate adaptation to the canal walls and resorptive defect. This technique aimed to provide a stable seal, promote healing, and maintain the structural integrity of the tooth. The quality of the bio-obturation was confirmed with an immediate postoperative radiograph, which showed proper filling/sealing and no obvious complications (Figure 2A). A similar clinical procedure was repeated for tooth #46 the next day (Figure 2B).

**Figure 3:**
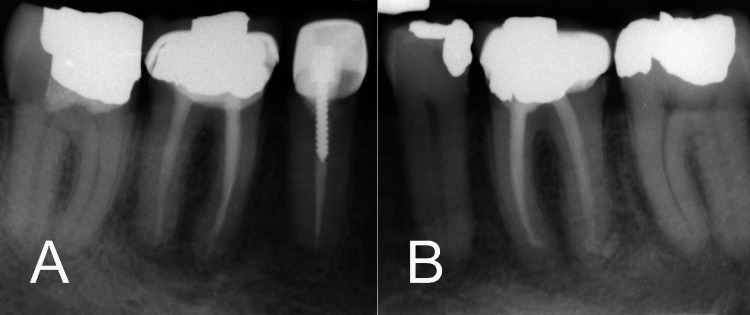
Five-year follow-up radiographs. (A) Tooth #36 showing complete resolution of apical radiolucency, reestablishment of a normal PDL, and absence of symptoms. (B) Tooth #46 revealing similar healing outcomes, with full resolution of the apical lesions and maintenance of the treated tooth structure. PDL: periodontal ligament

The treated teeth were closely monitored during follow-up visits. Within a week postoperatively, the patient reported complete resolution of symptoms, including the elimination of discomfort during chewing and absence of tenderness to percussion or palpation. The patient expressed satisfaction with the treatment, noting a significant improvement in overall oral comfort and functionality. At the three-month recall appointment, both teeth were functional and asymptomatic. Radiographic examination at the five-year follow-up revealed complete healing of the apical lesions in both treated teeth, with the reestablishment of a normal periodontal ligament, confirming the long-term success of the treatment (Figures 3A, 3B).

## Discussion

IRR is a pathological condition that can complicate endodontic treatment, particularly when it occurs in previously treated teeth. This case highlights the successful management of IRR in two molars with large apical lesions, treated nonsurgically using bio-obturation with biocompatible materials, specifically CEM cement. The diagnosis of IRR is often challenging, as it can be mistaken for other forms of resorption, such as external inflammatory resorption or cervical invasive resorption. In this case, the resorptive defects were localized to the apical region and associated with chronic inflammatory changes, which were clearly visualized through radiographic examination. The treatment approach was guided by the presence of these resorptive defects and the goal of preserving the natural tooth structure while promoting healing.

A key challenge in treating IRR is ensuring effective obturation, especially in cases with resorptive defects that disrupt the integrity of the canal walls. Traditional obturation techniques, such as gutta-percha with sealers, may not always provide a reliable seal in the presence of these defects, potentially leading to reinfection and treatment failure. In this case, the use of calcium silicate-based cement allowed for a more adaptable and biocompatible filling material, which has been shown to enhance sealing properties and promote tissue regeneration [8]. Studies have demonstrated that CEM cement’s biocompatibility and superior sealing ability make it particularly effective in managing complex resorptive defects and can lead to favorable long-term outcomes [8].

The nonsurgical retreatment approach, involving the use of bio-obturation techniques, offers several advantages. It preserves the existing PFM crowns, avoids the need for surgical intervention, and provides a stable internal seal that promotes the healing of the periapical tissues. This method is consistent with contemporary endodontic principles that emphasize the use of biocompatible endodontic materials to enhance the success of retreatment, particularly in cases where resorptive lesions are present [7]. Furthermore, the patient’s resolution of symptoms and the complete radiographic healing observed at the five-year follow-up demonstrate the efficacy of this approach.

In this case, the lack of significant postoperative complications and the long-term radiographic success align with existing literature supporting the use of endodontic biomaterials in managing IRR [4]. Recent studies have highlighted the increasing use of bio-obturation materials, such as MTA and CEM, due to their ability to create a durable seal and promote regeneration of periapical tissues. These biomaterials also reduce the risk of reinfection, which is a common concern in resorptive cases. While bioceramic cements offer benefits such as biocompatibility, antimicrobial properties, and tissue regeneration promotion, their relatively high cost, need for precise handling and advanced tools, extended setting time, and potential brittleness in highly stressed areas may limit their clinical applicability, particularly for nonsurgical retreatment of canals. For endodontists familiar with apical plug insertion techniques, bio-obturation may be considered a routine procedure, given the similarities in handling and the goal of achieving a secure and biologically compatible seal.

This study supports the notion that nonsurgical retreatment using biomaterials can be an effective treatment option for teeth affected by IRR, particularly when early diagnosis and appropriate biomaterials are utilized. However, further research and long-term clinical studies are needed to fully establish the success rates and outcomes of various biomaterials in the management of IRR, especially in more severe cases with extensive resorptive defects.

## Conclusions

This case emphasizes the effectiveness of bio-obturation, a nonsurgical retreatment approach using biocompatible endodontic materials like CEM cement, in managing IRR in endodontically treated teeth with large apical lesions and resorptive defects. Bio-obturation, with its focus on biocompatibility and tissue regeneration, provides a superior method of sealing the root canal system and promoting the healing of the periapical tissues, while preserving the natural tooth structure. The use of CEM cement in this case not only enhanced the sealing properties of the root canal but also facilitated tissue regeneration and minimized the risk of reinfection.

While the long-term clinical/radiographic success observed in this case supports the growing role of bio-obturation in treating complex cases of IRR, the study is inherently limited by its single-case nature and lack of long-term data from larger cohorts. Further research is needed to validate these findings in broader clinical settings, considering variations in patient demographics, treatment complexities, and long-term outcomes. Despite these limitations, nonsurgical retreatment using bio-obturation should be considered a viable and effective option for managing IRR, particularly when early diagnosis and appropriate biomaterials are utilized.
